# Elective neck dissection versus wait-and-see policy in cT1N0 buccal squamous cell carcinoma

**DOI:** 10.1186/s12885-020-07006-w

**Published:** 2020-06-09

**Authors:** Qigen Fang, Hua Gao, Qing Gao, Jinlan Sun, Peng Li, Meng Cui, Enxi Zhang, Wenlong Yin, Yuanyuan Dong

**Affiliations:** 1 Department of Head Neck and Thyroid, Affiliated Cancer Hospital of Zhengzhou University, Henan Cancer Hospital, Zhengzhou, Henan Province People’s Republic of China; 2 Department of Oral Medicine, Central Hospital of Yingkou, Yingkou, Liaoning Province People’s Republic of China; 3 Disease Control and Prevention Center, Shenyang, Liaoning People’s Republic of China; 4 Department of Oral Medicine, Kaifeng Central Hospital, Kaifeng, Henan People’s Republic of China

**Keywords:** Buccal squamous cell carcinoma, Elective neck dissection, Early-stage oral squamous cell carcinoma, Squamous cell carcinoma, Occult lymph node metastasis

## Abstract

**Background:**

Our goal was to clarify the comparison between elective neck dissection (END) and the wait-and-see policy in neck management for cT1N0 buccal squamous cell carcinoma (SCC).

**Methods:**

This was a retrospective comparison of 175 prospectively enrolled patients with cT1N0 buccal SCC. The patients were divided into two groups based on the nonrandomized management of the neck: 125 patients received END, and 50 patients were exposed to the wait-and-see policy. The main study endpoints were locoregional control (LRC) and disease-specific survival (DSS). Patients were asked to complete the shoulder domain in the University of Washington quality of life questionnaire, version 4, 1 year postoperatively.

**Results:**

Ten of the patients undergoing END developed recurrence, and the 5-year LRC rate was 92%. Five patients undergoing the wait-and-see policy developed recurrence, and the 5-year LRC rate was 90%. The difference was not significant (*p* = 0.668). There were 6 deaths in patients undergoing END, and the 5-year DSS rate was 94%. There were 3 deaths in patients undergoing the wait-and-see policy, and the 5-year DSS rate was 94%; the difference was not significant (*p* = 0.777). The mean shoulder scores of patients undergoing END and the wait-and-see policy were 93.9 and 100, respectively, and the difference was not significant (*p* = 0.284).

**Conclusion:**

Elective neck dissection does not carry a survival benefit compared to the wait-and-see policy, and it is not suggested for patients with cT1N0 buccal SCC.

## Background

Neck lymph node metastasis is one of the most important prognostic factors in head and neck squamous cell carcinoma (SCC) [1–3], and early detection of neck lymph node disease is important for achieving better survival. However, owing to the wide range of occult lymph node metastasis rates in cT1N0 buccal SCC [4–15], there is great controversy regarding the best neck management. Researchers who support routine neck dissection believe that elective neck dissection (END) is able to identify patients who need adjuvant treatment and provide better survival, but a number of scholars insist that there is a great deal of overtreatment in patients without pathologic neck lymph node metastasis based on the relatively low metastasis rate of cT1N0 buccal SCC. Therefore, in the current study, we aimed to clarify the comparison between END and the wait-and-see policy in neck management for cT1N0 buccal SCC by a prospective study.

## Methods

The Zhengzhou University institutional research committee approved our study, and all participants provided written informed consent for medical research prior to initial treatment, and all experiments were performed in accordance with relevant guidelines and regulations.

Patients with cT1N0 buccal SCC based on the 7th AJCC staging system during January 2008 and December 2018 were prospectively enrolled. Staging of the neck was evaluated by palpation combined with routine contrast-enhanced CT and MRI [7, 8]. Surgery was the first-line treatment for every patient in our department, and the decision of END was mainly based on interviews between the surgeon and the patient. We clearly explained the difference between END and the wait-and-see policy according to at least three aspects: survival benefits, surgical morbidity, and economic requirements. All pathologic sections were reviewed by at least two pathologists. Perineural invasion (PNI) was considered to be present if tumor cells were identified within the perineural space and/or nerve bundle; lymphovascular infiltration (LVI) was positive if tumor cells were noted within the lymphovascular channels [16, 17]. The depth of invasion was measured from the level of the adjacent normal mucosa to the deepest point of tumor infiltration, regardless of the presence or absence of ulceration. The indications for adjuvant radiotherapy included neck lymph node metastasis, PNI, LVI, and positive margins. For a pN0 neck, the radiation field included ipsilateral levels I to III, and for a pN+ neck, the radiation field included ipsilateral levels I to V.

The operation was performed under general anesthesia, and END of ipsilateral levels I-III was first conducted if the patient agreed. Complete cancer resection with at least a 1-cm margin was required in all patients. The primary defect was reconstructed with primary closure, a skin graft, a biomembrane, or a pedicled flap.

Data regarding age, sex, TNM stage, pathologic characteristics, adjuvant treatment and follow-up were collected and analyzed. The patients were followed every 3 months in the first 2 years, every 6 months in the third to fifth years, and every 1 year from the fifth year thereafter. If recurrent disease was suspected, immediate interference was performed. During the follow-up, the patients were asked to complete the domain of shoulder function in the University of Washington quality of life questionnaire, version 4, 1 year postoperatively. The shoulder domain had four response options that were scaled evenly from 0 (worst) to 100 (best).

The demographic information between the two groups was compared with the Chi-square test or Student’s t test. The main research endpoints were locoregional control (LRC) and disease-specific survival (DSS). The survival times associated with LRC and DSS were calculated from the date of surgery to the date of the first locoregional recurrence or the cancer-related death, respectively. The Kaplan-Meier method was used to calculate the survival rate. A Cox model was used to determine the independent prognostic factors. A nonparametric Mann-Whitney U test was used to analyze the shoulder domain score. All statistical analyses were performed using SPSS 20.0, and *p* < 0.05 was considered to be significant.

## Results

During our study period, a total of 175 patients were enrolled; there were 132 males and 43 females, with a mean age of 48.4 (range: 25–68) years. There were 125 patients who underwent END, and 50 patients were exposed to the wait-and-see policy. Negative margins were achieved in all patients. In patients undergoing END, the mean number of dissected lymph nodes was 24.3 (range: 10–40), and positive lymph nodes were noted in 10 patients: 8 patients had one positive lymph node at level I, and 2 patients had one positive lymph node at level I and one positive lymph node at level IIa.

The demographic and pathologic information of the two groups is compared in Table 1. No cT1 tumors were reclassified as pathologic T2 tumors. No patients received adjuvant chemotherapy, and the two groups had no apparent difference regarding age, sex, smoking status, drinking status, educational background, economic status, invasion of depth, PNI, LVI, tumor differentiation, adjuvant treatment, or follow-up time. The mean hospitalization expenses were 26,000 RMB and 22,000 RMB for patients undergoing END and the wait-and-see policy, respectively, and the difference was significant (*p* < 0.001).

**Table 1:** Comparison of demographic and pathologic data between the two groups

Variables	Elective neck dissection (*n* = 125)	Wait-and-see policy (*n* = 50)	*p*
Age			
<40	10	6	
≥40	115	44	0.562
Sex			
Male	93	39	
Female	32	11	0.617
Smoker			
Yes	75	37	
No	50	13	0.081
Alcohol Use			
Yes	63	28	
No	62	22	0.503
Education			
<High school	35	10	
≥High school	90	40	0.274
Area			
Countryside	89	29	
Urban	36	21	0.092
Hospitalization expenses	26,000 (24000–30,000)	22,000 (18000–26,000)	< 0.001
PNI^a^			
Yes	10	4	
No	115	46	1.000
LVI^&^			
Yes	7	4	
No	118	46	0.731
Invasion depth (mm)	2.4 (1.7–4.7)	2.3 (1.8–4.6)	0.584
Tumor differentiation			
Well	50	18	
Moderate	68	29	
Poor	7	3	0.925
Radiotherapy			
Yes	15	8	
No	110	42	0.453
Follow-up time	59.4 (7–115)	64.0 (6–105)	0.226

^a^*PNI* perineural invasion, *LVI* lymphovascular infiltration

A total of 109 patients undergoing END and 40 patients undergoing the wait-and-see policy completed the questionnaire, and the mean shoulder scores of patients undergoing END or the wait-and-see policy were 93.9 and 100, respectively; the difference was not significant (*p* = 0.284).

**Fig. 1:**
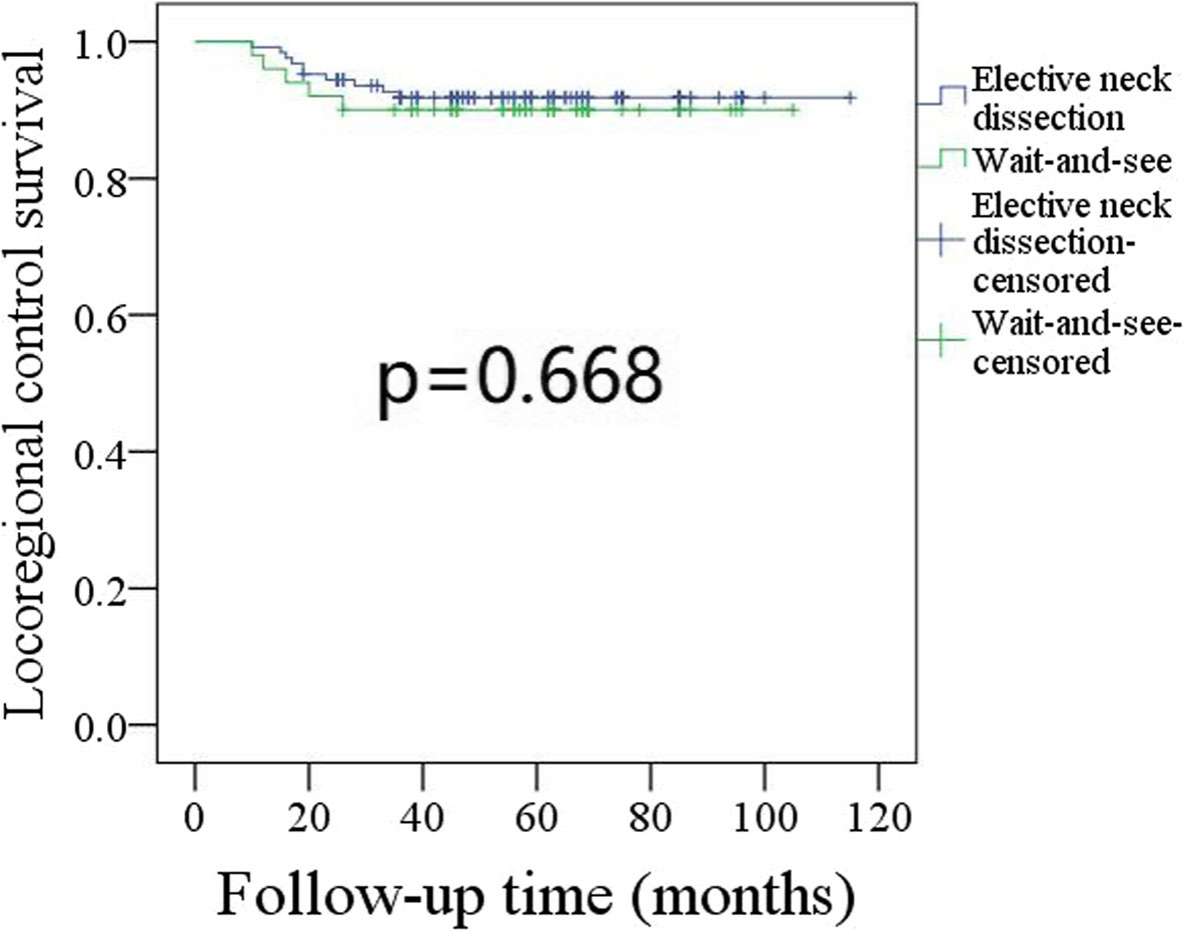
Comparison of locoregional control survival in patients undergoing elective neck dissection or wait-and-see policy (*p* = 0.668)

During our follow-up with a mean time of 60.7 (range: 6–115) months, in patients undergoing END, 15 (12.0%) patients received adjuvant radiotherapy, and 10 (8.0%) patients developed recurrence: local recurrence occurred in 2 (20.0%, 2/10) patients, and regional recurrence occurred in 8 (80.0%, 8/10) patients: ipsilateral lymph node recurrence in level I, II, III, IV, and V occurred in 2, 3, 2, 1, and 1 patients, respectively; contralateral lymph node recurrence in level I, II, and III occurred in 1, 1, and 1 patient, respectively (Table 2). Four patients underwent successful salvage operations, and the 5-year LRC rate was 92%. In patients undergoing the wait-and-see policy, 8 (16.0%) patients received adjuvant radiotherapy, and 5 (10.0%) patients developed recurrence: local recurrence occurred in 1 (20.0%, 1/5) patient, and regional recurrence occurred in 4 (80.0%, 4/5) patients: ipsilateral lymph node recurrence in level I, II, and III occurred in 2, 2, and 1 patients, respectively; contralateral lymph node recurrence in level Ioccurred in 1 patient (Table 2). Two patients underwent successful salvage operations, and the 5-year LRC rate was 90%. The difference was not significant (Fig. 1, *p* = 0.668).

**Table 2:** Neck recurrence pattern in the two groups

Level	Elective neck dissection (*n* = 8)		Wait-and-see policy (*n* = 4)	
	Ipsilateral	Contralateral	Ipsilateral	Contralateral
I	2	1	2	1
II	3	1	2	0
III	2	1	1	0
IV	1	0	0	0
V	1	0	0	0

**Fig. 2:**
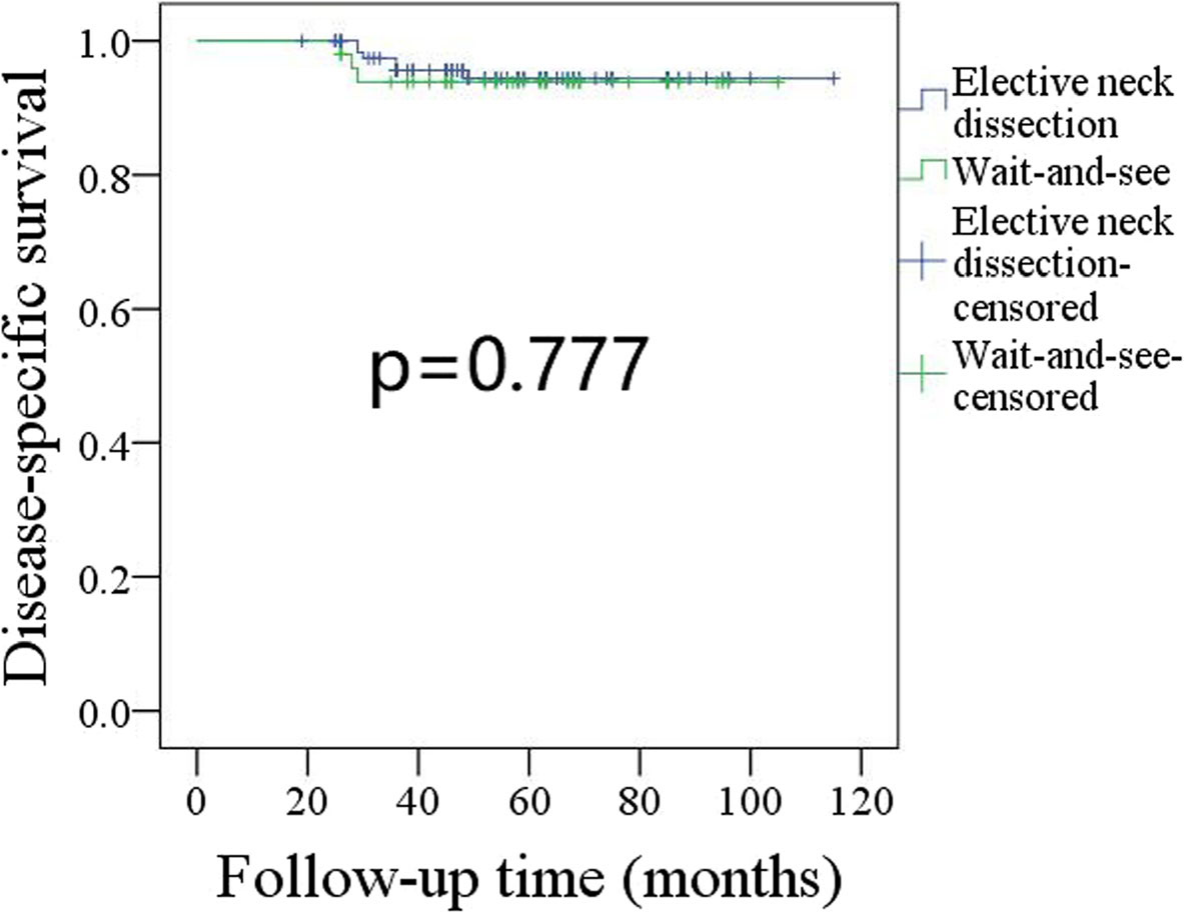
Comparison of disease-specific survival in patients undergoing elective neck dissection or wait-and-see policy (*p* = 0.777)

There were 6 deaths in patients undergoing END, and the 5-year DSS rate was 94%. There were 3 deaths in patients undergoing the wait-and-see policy, and the 5-year DSS rate was 94%; the difference was not significant (Fig. 2, *p* = 0.777).

## Discussion

The main finding in the current study was that END led to similar LRC and DSS rates compared to the wait-and-see policy, and END was similar to the wait-and-see policy regarding shoulder function. However, there was a higher hospitalization cost associated with END than with the wait-and-see policy.

A number of authors have aimed to clarify the role of END in the treatment of buccal SCC. Dillon et al. [6] found that the 2- and 5-year survival rates for N0 patients without END were 80 and 40%, respectively, vs 93 and 87% for those who underwent END (*p* = 0.002), and the authors concluded that END had a therapeutic role in increasing disease control. However, the study only enrolled 20 subjects. Cariati et al. [4] showed that the high risk of local recurrence was associated with protection of the neck from future cervical recurrence, even in small T1 tumors, and from supraomohyoid neck dissection, even in cT1N0 disease, if there was suspicion that the tumor thickness was greater than 4 mm. A similar viewpoint was also supported by Lubek et al. [18]. However, all these studies were retrospective, and they included stage T1-T4 cancer. Both Hakeem et al. [8] and Iyer et al. [9] noted that the occult node metastasis rate was less than 20% for patients with T1 buccal SCC, and these authors did not recommend routine END. The same proposal was also supported by Dhawan et al. [19], but all these authors did not compare the outcomes of patients who did or did not undergo END. Huang et al. [20] previously compared the oncologic outcomes between 151 cT1–2 N0 buccal SCC patients with END and 22 patients who underwent a wait-and-see policy; the authors noted that patients undergoing END had better neck control, but the two groups had similar overall survival. This interesting finding could be explained by the fact that the metastatic lesions were mostly salvageable in patients exposed to the wait-and-see policy, and the importance of regular follow-up was emphasized. In the current study, the occult lymph node metastasis rate was 8%, a finding that was consistent with previous reports [8, 9, 19]. Moreover, we found that END did not carry a survival benefit compared to the wait-and-see policy, and this finding was important. Similar literature regarding tongue SCC is extensive and usually suggests routine END in T1 tongue SCC [21–23], but the survival and biologic behavior show significant differences between buccal SCC and tongue SCC [24]; buccal SCC might have unique characteristics, and our clinical outcome confirmed this hypothesis.

Shoulder dysfunction is not an uncommon complication after neck dissection. Chan et al. [25] recruited 46 patients undergoing neck dissection for recurrent nasopharyngeal carcinoma, and the authors found that the mean Disability of Arm, Shoulder, and Hand score was 44.2 in the first year posttreatment, and the mean Disability of Arm, Shoulder, and Hand score was 46.3 in the second year posttreatment. The degree of daily activity affected was rated as moderate to very limited, and 30% of the patients had at least moderate shoulder pain at rest; the authors concluded that shoulder dysfunction after neck dissection was significant and persistent. Similar reports have also been described by Watkins et al. [26], but Teymoortash et al. [27] insisted that neck dissection-related complications arose in only two patients with an incidence of 4%, and neck dissection showed a low incidence of surgical complications and acceptable functional and esthetic results. In our study, we noted that only 13.8% of the patients undergoing END complained of shoulder dysfunction subjectively, and the dysfunction was minor; therefore, the two groups had similar results. However, we must keep in mind that shoulder dysfunction was significantly associated with the operation time, preservation of the accessory nerve, dissection type, and adjuvant radiotherapy.

Owing to the different types of medical insurance in our countries, economic status had a significant effect on the treatment decision, especially in patients from low-income families. However, it was noted that there were higher hospitalization expenses in patients undergoing END than in patients exposed to the wait-and-see policy, and more rural patients received END. There are at least three aspects that could help to comprehend our findings: first, the additional surgical procedure associated with neck dissection certainly increased the expenses; second, the high expenses required by routine follow-up associated with the wait-and-see policy were not calculated in the current study; and third, routine follow-up was difficult to implement in some patients because the patients with oral SCC usually came from remote areas, and the follow-up time for rural patients was significantly shorter than that for patients from urban areas (55.3 months vs 72.0 months, *p* < 0.001).

Limitation of current study must be acknowledged: the sample size was relatively small, it could decrease the power ability; this study was short of randomization, then there was significant selection bias, and more high-quality studies are needed.

## Conclusions

In summary, elective neck dissection does not have a survival benefit compared to the wait-and-see policy, and it is not suggested for patients with cT1N0 buccal SCC.

## Supplementary information

**Additional file 1: Supplementary Figure 1**. Locoregional control survival in patients with different depth of invasion (*p* = 0.016).

**Additional file 2: Supplementary Figure 2**. Disease specific survival in patients with different depth of invasion (*p* = 0.006).

## Data Availability

All data generated or analyzed during this study are included in this published article. And the primary data could be achieved from the corresponding author.

## Abbreviations

SCC: Squamous cell carcinoma
END: Elective neck dissection
LRC: Locoregional control survival
DSS: Disease specific survival
