# Supplementary figures and images for: Elective neck dissection versus wait-and-see policy in cT1N0 buccal squamous cell carcinoma
Source: BMC Cancer. 2020 Jun 9;20:537. doi: 10.1186/s12885-020-07006-w (PMC7285468; doi:10.1186/s12885-020-07006-w)

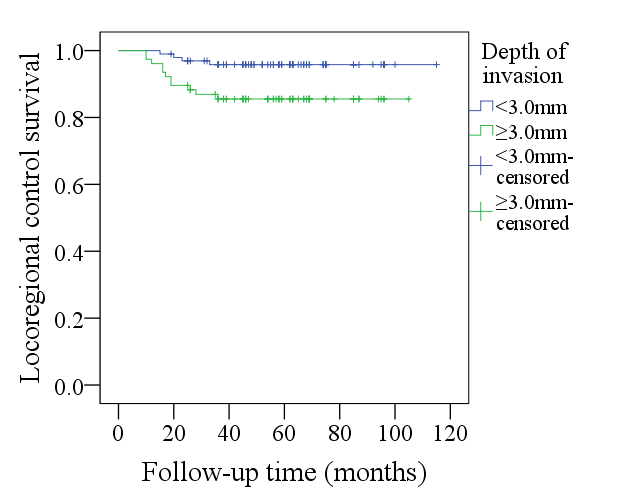

Supplement: Supplementary file 1 — Additional file 1: Supplementary Figure 1. Locoregional control survival in patients with different depth of invasion (p = 0.016). [file 12885_2020_7006_MOESM1_ESM.tif]

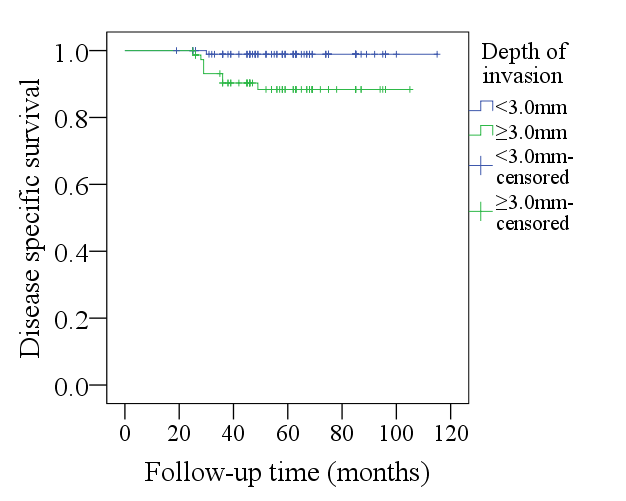

Supplement: Supplementary file 2 — Additional file 2: Supplementary Figure 2. Disease specific survival in patients with different depth of invasion (p = 0.006). [file 12885_2020_7006_MOESM2_ESM.tif]
